# Leveraging Neural Crest-Derived Tumors to Identify NF1 Cancer Stem Cell Signatures

**DOI:** 10.3390/cancers16213639

**Published:** 2024-10-29

**Authors:** Sajjad Khan, Donia Alson, Li Sun, Caroline Maloney, Daochun Sun

**Affiliations:** 1 Department of Cell Biology, Neurobiology and Anatomy, Medical College of Wisconsin, Milwaukee, WI 53226, USA; 2 Department of Pediatric Surgery, Medical College of Wisconsin, Milwaukee, WI 53226, USA; 3 Cancer Center, Medical College of Wisconsin, Milwaukee, WI 53226, USA; 4 Department of Pediatric, Medical College of Wisconsin, Milwaukee, WI 53226, USA; 5 Children Research Institute, Milwaukee, WI 53226, USA

**Keywords:** NF1, neural crest stem cell, cancer stem cell, gene signatures, CD44

## Abstract

Neurofibromatosis type 1 (NF1) is a genetic disorder that predisposes individuals to develop benign and malignant tumors of the nerve sheath. Understanding the signatures of cancer stem cells (CSCs) for NF1-associated tumors may facilitate the early detection of tumor progression. **Background:** Neural crest cells, the cell of origin of NF1-associated tumors, can initiate multiple tumor types, including melanoma, neuroblastoma, and schwannoma. CSCs within these tumors have been reported; however, identifying and targeting CSC populations remains a challenge. **Results:** This study aims to leverage existing studies on neural crest-derived CSCs to explore markers pertinent to NF1 tumorigenesis. By focusing on the molecular and cellular dynamics within these tumors, we summarize CSC signatures in tumor maintenance, progression, and treatment resistance. **Conclusion:** A review of these signatures in the context of NF1 will provide insights into NF1 tumor biology and pave the way for developing targeted therapies and improving treatment outcomes for NF1 patients.

## Simple Summary

Understanding neural crest stem cells as the cells of origin for a series of tumors expands the potential strategies for treatment, especially for rare tumor types. The shared properties and signatures of stem-like tumor cells, also called cancer stem cells (CSCs), are reviewed for neural crest-originated tumors. Scrutinizing the relationship between neural crest stem cells and CSCs in NF1-associated tumors can allow for earlier detection and treatment in NF1 patients. Previously identified CSC markers can be the key to NF1 tumor pathogenesis and therapeutic targets. Targeting CD44, for example, can potentially reduce CSC-related tumor progression, relapse, and even metastasis. This review delineates the reported CSCs in tumors of neural crest origin and highlights their potential relevance in NF1-associated tumors.

## 1. Introduction

Neurofibromatosis type 1 (NF1) is a complex genetic syndrome prominently characterized by the development of multiple peripheral nerve-associated neurofibromas. These tumors originate from the early Schwann cell lineage (SCL), which is derived from neural crest stem cells (NCSCs). These tumors can develop early in the life of NF1 patients and continue to progress over their lifetime [1,2]. The neural crest lineage is critical during embryonic development to give rise to a large variety of cell types. This pluripotency predisposes NCSC-derived tissues to a broad range of tumors with different driver mutations, including melanoma, neuroblastoma, and various types of sarcomas and neurofibromas [3]. These tumors share neural crest molecular signatures, such as the Schwann cell progenitor markers SOX10, P75 (NGFR), and S100b [4,5]. Although the incidences of these tumors are relatively low in the general population, neural crest-derived tumors (NCDTs) disproportionately impact pediatric patients. Understanding the cell of origin and studying the common features across different NCDTs are essential for closing critical knowledge gaps and developing better treatment strategies for NF1 tumors.

The evidence that tumors can arise from cells that retain stem cell-like properties has led to the cancer stem cell (CSC) hypothesis. Stem cell-like characteristics can contribute to cancer initiation, progression, resistance to therapy, and metastasis [1,6]. The concept of CSC continuously evolves and significantly expands the understanding of hematopoietic malignancy as well as solid tumors. It was recently reported that NCDTs harbor stem-like cells bearing properties similar to NCSCs [4,7]. The identification of CSC molecular signatures may enable early detection of malignant progression in neurofibromas, improve monitoring techniques, and lead to the development of targeted therapies to prevent malignant transformation. This review aims to compare and summarize the gene signatures of stem-like cells in NCDTs, thereby providing insights into potential biomarkers and therapeutic targets for NF1-related tumors.

## 2. Neural Crest Origin and Oncogenic Potential in NF1 Tumors

The neural crest is a transient embryonic structure in vertebrates that plays a critical role during early development. A recent study indicates that the OCT4-SOX2 dimer regulates the epigenomic landscape of the neural crest and differentiates these cells from embryonic stem cells [8]. NCSCs are collections of pluripotent stem cells that arise from the neural tube and non-neural ectoderm border during neurulation and migrate throughout the body. There are controversial opinions on whether NCSCs are precursors with restricted fate in development [9,10]. However, it is well known that NCSCs can differentiate into various cell lineages, including melanocytes, craniofacial cartilage and bone, smooth muscle, peripheral and enteric neurons, and glia [11]. Their pluripotency and migratory abilities have made NCSCs a point of interest in cancer research. The regulatory pathways for NCSC differentiation, proliferation, survival, and migration are tightly orchestrated during embryogenesis. Meanwhile, these regulatory signals, such as WNT, Notch, Hedgehog, STAT3/5, and RAS/MAPK/AKT, are often found to be aberrantly activated in a variety of tumors [12,13,14,15,16]. Therefore, these conserved markers of NCSC development may highlight critical vulnerabilities in forming NCDTs. Additionally, NCSCs may have relatively low immunogenicity due to their migratory properties during development, which may provide a survival advantage in evading the immune system [17]. Targeting these NCSC pathways offers a strategic approach to disrupt the malignant progression of these tumors and may lead to more effective therapy to block tumorigenesis. Exploring and exploiting these vulnerabilities remain a promising frontier in treating these complex and diverse tumors.

NF1 is a monogenic disorder influencing 1:2500 newborns each year. NF1 patients are susceptible to multiple disorders, including the growth of benign and malignant tumors associated with the central and peripheral nervous systems. Cutaneous neurofibromas (cNFs) and plexiform neurofibromas (pNFs) are benign tumors, but the latter has a higher risk of progression to malignant peripheral nerve sheath tumors (MPNSTs). To date, no targeted treatment for MPNSTs has been successful. Stem-like tumor cells have been reported to promote tumor progression and drug resistance in NF1-associated tumors [2]. Genetically engineered mouse models (GEMMs) of pNFs and MPNSTs utilize promoters of neural crest or early Schwann cell progenitor genes, such as *Krox20*, *Hoxb7*, *Dhh*, and *Prss56*, and provide solid support of NF1 tumor initiation and demonstrate their neural crest origin [18,19,20]. NCSC markers, including but not limited to *Sox10*, *ErbB3*, and *Nes*, are highly expressed in CSCs in NF1-associated tumors in both human and mouse studies.

The CSC theory argues that tumors develop in a hierarchy at the apex of which sits a unique set of stem-like cells that are responsible for giving rise to the rapidly proliferating tumor cells and serve as conveyors of drug resistance and tumor re-emergence following therapy [5,21]. Having hijacked the essential stem cell properties of cell cycle regulation and DNA damage repair, NF1-associated tumors have a relatively low mutation burden [2,22,23]. Conserved NCSC developmental signatures can be identified across a variety of NCDTs, such as activated RAS signaling and loss function of CDKN2A; however, they manifest at different development stages according to individual mutations and tissue types. This commonality favors the extension of such NCSC signatures to lesser-known NCDTs to explore potential vulnerabilities.

## 3. Characterization of CSCs in NF1 Tumor Models

Recently, studies have explored the presence and role of CSCs in NF1-associated tumors. CD133 (Prominin-1), a glycoprotein often expressed on stem-like cells from many cancer types, has been proposed as a CSC marker in MPNSTs [24]. In culture and transplantation, CD133-positive spheres are more tumorigenic than CD133-negative cells and display elevated RAS signaling and resistance to ERK inhibition [25]. However, these studies only examined limited MPNST cell lines. When examining NF1 GEMMs, *Krox20Cre;Nf1^f/−^* targets NCSCs and leads to the generation of NF1 tumors [18]. Kershner et al. reported the presence of stem-like tumor cells within the pNFs generated by the *DhhCre;NF1^f/f^* model, and a further enriched subpopulation using NGFR+&EGFR+ in cell sorting can form tumors in transplantation [26]. This *Dhh* promoter turns on as early as embryonic day 12.5 (E12.5) [27] and targets specific NCSC populations and derivatives within the embryonic dorsal root ganglia (DRG). In the recent *Prss56Cre;NF1^f/f^* model, Radomska et al. described the tumorigenesis occurring from the E13.5 DRG and boundary cap cells [20]. Interestingly, the *Prss56Cre;NF1f/f* model can potentially recapitulate the full spectrum of NF1 tumorigenesis, including cNFs, pNFs, and MPNSTs [28]. Using a transgenic mouse model with enhanced GFP and rat *Nes* Cre, Sun et al. identified a GFP-positive population at both E13.5 DRG and MPNSTs with the *Nf1^f/f^;Trp53^f/f^* configuration driven by rat *Nes* promoter and its 2nd intron [2,29,30]. The sorted GFP-positive tumor cells demonstrated similar NCSC signatures, including *ERBB3*, *SOX10*, and *NES*, as seen in both mouse tumors and human low-grade MPNSTs [2]. Using single-cell RNA sequencing (scRNAseq) technology, Wu et al. identified a mouse *Nes*-negative, mesenchymal stem-like population in the *DhhCre;ccGFP;Nf1^f/f^;Trp53^f/f^;Eed^f/f^* model and demonstrated that this population could functionally promote MPNST tumorigenesis [31]. Zhang et al. also characterized a stem-like oncogenic transcriptional program in human MPSNTs and suggested loss of PRC2 may sustain this population using scRNAseq on paired primary and metastasis MPNSTs [32]. These studies provide ample evidence of the stem cell-like population in NF1 tumors and its essential role in promoting malignant transformation. Further identification of specific CSC markers within this population of cells may enable early detection and intervention to prevent NF1-associated tumor transformation and metastasis.

## 4. CSC Signatures of NCDTs

### 4.1. Melanoma CSC Signatures

**Figure 1:**
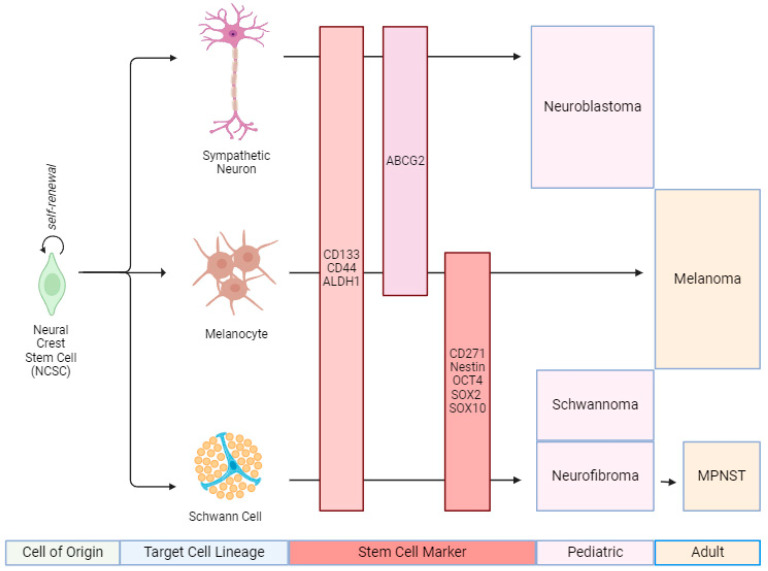
NCSC-derived tumor types with common signatures. A schematic graph shows the NCSC lineages and the reported markers for respective tumor types.

Melanoma is a tumor that arises from the melanocyte lineage. Melanoblasts derived from E12.5 to E16.5 NCSCs and Schwann cell progenitors can give rise to melanocytes in the skin (Figure 1) [33,34]. Melanoma is one of the most aggressive forms of skin cancer, characterized by a high propensity for metastasis and resistance to conventional therapies. Its etiology involves a complex interplay of genetic and environmental factors. Ultraviolet radiation is a well-established risk factor, resulting in DNA damage and subsequent mutations in key oncogenes and tumor suppressor genes, such as *BRAF*, *NF1*, *CDKN2A*, and *TRP53* [35]. The aggressiveness of melanoma can be attributed to a high degree of tumor cell plasticity, mirroring the multipotency of early progenitor cells. CSC populations in melanoma have been widely studied. Tumor cells harboring CSC markers (e.g., CD133 [36] and ABCB5 [37]) possess the ability to form tumors in immunodeficient mice at a significantly lower cell number compared to non-CSC populations [36,38]. These CSCs often show resistance to chemotherapy and radiotherapy [39].

CD271 is involved in regulating neural crest cell migration and cell fate. In melanoma, it serves as a molecular switch, enabling the rapid and reversible conversion between proliferative and invasive or stem-like and non-stem-like states [40]. The expression of CD271 is associated with metastatic progression and resistance to chemotherapeutic agents [41,42]. Interestingly, NF1-associated pNFs and MPNSTs also express CD271, suggesting a mechanism wherein stem-like properties may contribute to tumor progression [24].

CD44 is a cell surface glycoprotein that has been identified as a CSC marker in melanoma. This transmembrane protein is involved in cell–cell interactions, cell adhesion, and migration, making it an important marker in tumor progression and metastasis [43]. Harwood et al. demonstrated dynamic CD44 expression in different melanocytic lesions [44]. CD44 interacts with components of the extracellular matrix, such as hyaluronic acid, facilitating not only migration but also invasion of melanoma cells [45,46].

### 4.2. Neuroblastoma CSC Signatures

Neuroblastoma is a type of pediatric malignant tumor that arises from the sympathetic nervous system [47]. Neuroblastomas typically grow in the adrenal medulla or paraspinal ganglia. CSCs in neuroblastoma have been extensively studied [48]. Neuroblastoma is characterized by the heterogeneous expressions of SOX10 and PHOX2B, which are essential for NCSC differentiation [49]. The presence of CD133 and ALDH1 in neuroblastoma also highlights a subpopulation with stem-like features, capable of initiating tumors and resisting conventional treatments [50]. Similar to neuroblastoma, ALDH1 expression has been reported in NF1-associated tumors [51]. The decreased ALDH1 immunostaining from pNFs to MPNSTs and variable expression pattern in MPNST tissues suggest heterogeneous populations that may drive tumor progression and complexity. Additionally, studies have shown that CD44-expressing neuroblastoma cells exhibit higher tumorigenic potential and are involved in maintaining the stemness of tumor cells [52]. Mehrazma et al. evaluated CD44 expression in pediatric solid tumors, including neuroblastoma, and suggested that CD44 prognostic value may vary across different cancer types [53]. Jensen et al. showed that metastatic neuroblastoma was characterized by an increased expression of the CD44 marker, compared to non-metastatic controls [54]. CD44-expressing CSCs are associated with tumor aggressiveness and poor survival in neuroblastoma [52].

### 4.3. Schwannoma CSC Signatures

Schwannomas are typically encapsulated and slow-growing. They can occur sporadically or in association with genetic conditions like Neurofibromatosis Type 2 [55]. While schwannomas are generally benign, they can cause significant morbidity due to their location and size, leading to nerve compression and associated symptoms. Markers such as S100, GFAP, and SOX10 are commonly used to identify these tumors [56]. Studies from Kilmister et al. demonstrated that cell subpopulations within schwannomas express embryonic stem cell-like markers, including CD133 and Yamanaka factors [57]. The loss of function of the *NF2* gene, as a driver mutation, leads to deregulation of the Hippo pathway and RAS/MAPK signaling and further promotes tumor growth [58]. The overlapped markers and pathways in both schwannomas and NF1-related tumors underscore shared vulnerabilities that can be therapeutically exploited. Both tumors demonstrate a critical reliance on pathways regulating cytoskeletal dynamics and cellular contacts, highlighting a potential weakness of NCDTs. The common molecular signatures shared by CSCs in NCDTs are summarized in Table 1.

## 5. The Role of CD44 in NF1 Tumors

CD44, as a cell surface glycoprotein, has been reported in multiple tumor types as a marker for stem-like tumor cell populations [76]. CD44 can stimulate different signaling pathways vital to cell proliferation, cell death signaling blockade, epithelial–mesenchymal transition (EMT), and multidrug resistance. The roles of CD44 have been widely explored in a variety of cancers for tumor progression, metastasis, chemoresistance, and angiogenesis [77,78,79,80].

As a CSC marker, CD44 has been reported in breast cancer, colorectal cancer, pancreatic cancer, and head and neck squamous cell carcinoma [76]. For example, in colorectal cancer, CD44-expressing cells exhibit high tumorigenic capacity and are capable of initiating tumor formation in xenograft models [81]. Similarly, in pancreatic cancer, CD44-expressing CSCs are implicated in resistance to standard chemotherapy regimens [82].

In NCDTs, increasing evidence indicates that CD44-high cells with stem-like properties contribute significantly to tumor heterogeneity and therapeutic resistance (Table 1). The emerging research of CD44 in NF1 offers glimpses into the potential significance of tumorigenic processes associated with NF1. Considering the spatiotemporal characteristics of CD44 expression is crucial for understanding its biological significance. Sherman et al. demonstrated that CD44 expression during Schwann cell proliferation is higher in early rat neonatal nerves than in adult nerves and CD44 was found in some non-myelinating Schwann cells and varied in myelinating fibers [83]. The correlation of CD44 and enhanced malignancy has also been established using in vitro models. In the MPNST cell lines ST88-14 and 90-8, Su et al. established that increased CD44 expression correlated with increased malignant potential [84]. They further reported that high CD44 expression was mediated through the Src kinase pathway rather than the prevailing concept of mitogen-activated protein kinase (MAPK) activity. In vivo, Riddle et al. studied 28 benign neurofibromas and 16 MPNST human specimens obtained from NF1 patients to test for CD44 and p53 expressions, and the immunoexpression of CD44 was focally positive in all the tested pNF/cNF samples and was particularly upregulated in MPNSTs [85]. Moreover, Friedrich and coworkers studied the expression patterns of CD44 in samples obtained from 385 patients with NF1 tumors exhibiting both benign and malignant phenotypes [86]. Using immunochemistry, they determined the presence of CD44 in all samples and a pattern with significantly higher expression in diffusely growing tumors compared with the encapsulated pNFs. These findings further strengthen CD44’s roles in NF1 tumor progression and invasion.

CD44 has also been shown to govern neural stem cell properties. Su et al. found that CD44-expressing neural stem cells have the ability to differentiate into neurons, astrocytes, and oligodendrocytes in an autonomous manner [87]. They also determined that the quiescence of neural stem cells is regulated by the interaction of CD44 with hyaluronic acid (HA). The role of CD44-related interactions, resultant signaling cascades, and respective outcomes in different types of tumors are reviewed in detail by Skandalis et al. [88]. More importantly, CD44 is constitutively associated with ERBB2 and ERBB3 receptor tyrosine kinases, which heterodimerize in the cell membrane of Schwann cells in response to neuregulin. ERBB3 is a known neural crest signature that regulates NCSC differentiation. CD44 significantly enhances neuregulin-induced ERBB2/ERBB3 heterodimerization and, therefore, maintains the stem-like properties for CSCs in NF1 tumors [83].

In addition, HA–CD44 interactions have been previously shown to increase resistance to several drugs in various tumor types [89,90,91,92]. A similar effect on chemoresistance has been demonstrated in NF1-derived tumors. Slomiany et al. demonstrated a decrease in resistance to doxorubicin using both in vitro and in vivo cancer models owing to CD44 activity [93]. Their findings suggested complex interactions among CD44, multidrug transporters, P-glycoprotein, and breast cancer resistance protein mediated by hyaluronan (HA). By replacing HA with HA oligosaccharides, the CD44 drug transporter complex separated after the internalization of the multidrug transporters, which consequently improve doxorubicin sensitivity in MPNSTs.

## 6. Discussion

Recent advancements in cancer research have highlighted the pivotal role of CSCs in tumor initiation, progression, and therapeutic resistance. Their identification and characterization in NF1-associated tumors may revolutionize early detection and therapeutic approaches, particularly for early-onset pediatric pNFs. Early detection of CSCs enables timely intervention, which is crucial in preventing the progression of benign neurofibromas to MPNSTs. Markers identified in other NCDTs, such as melanoma and schwannomas, provide valuable insights into CSCs in NF1-associated tumors. Further investigation into markers such as CD44, NGFR, and ERBB3 may reveal their roles as CSC markers in maintaining stemness, promoting metastasis, and conferring resistance to therapy in NF1-associated tumors. Therapies, such as the inhibition of CD44-HA interactions, could potentially reduce stem-like properties, limit tumor growth, and enhance the efficacy of existing treatments [31,32].

Revealing the normal function of CSC markers in cellular crosstalk in NCSC development is essential for understanding tumorigenesis. For instance, CD44-facilitated ERBB2/3 heterodimer formation serves as a mechanism that has been reported to mediate metastasis in breast cancer models [94]. Since ERBB family receptors play an important role in SCL development, the correlation between ERBB receptors and CD44 in pNF development and progression to MPNSTs presents promising research questions. Similarly, understanding the spatiotemporal sequence of NF1 and CD44 dysregulation can increase our understanding of the pathogenesis and development of pNF and its progression. Utilizing these CSC markers to establish new targets would enable us to establish the CD44-targeting strategies for pNFs and MPNSTs.

## 7. Conclusions

By identifying and analyzing common CSC signatures across NCDTs, we gain insight into the mechanisms of tumor maintenance, progression, and treatment resistance. Specifically, targeting CSC markers in NF1 tumors presents a promising therapeutic strategy to inhibit tumorigenesis and improve treatment outcomes in NF1. Future research should focus on these markers to develop early detection methodology and targeted therapies to enhance personalized treatment and quality of life for patients.

## Figures and Tables

**Table 1:** Common CSC signatures among the NCDTs.

CSC Marker	NCDT	Reference
CD133 (Prominin-1)	Neuroblastoma, Melanoma, Schwannoma, MPNST	[2,36,59,60]
CD44	Neuroblastoma, Melanoma, Schwannoma, MPNST	[52,60,61,62]
Nestin	Melanoma, Neurofibroma, MPNST	[1,2,63]
ALDH1 (Aldehyde Dehydrogenase 1)	Neuroblastoma, Melanoma, Schwannoma, Neurofibroma, MPNST	[51,64,65]
SOX2	Neuroblastoma, Schwannoma, Melanoma	[57,66,67]
CD271 (p75NTR)	Melanoma, Schwannoma, Neurofibroma, MPNST	[2,42,68,69]
ABCG2 (BCRP1)	Neuroblastoma, Melanoma	[70,71]
OCT4	Neuroblastoma, Melanoma, Schwannoma	[57,72,73]
SOX10	Melanoma, Neurofibroma, MPNST	[2,74,75]
